# Taxonomic study on Japanese *Salvia* (Lamiaceae): Phylogenetic position of *S. akiensis*, and polyphyletic nature of S. lutescens var. intermedia

**DOI:** 10.3897/phytokeys.80.11611

**Published:** 2017-06-05

**Authors:** Atsuko Takano

**Affiliations:** 1 Division of Natural History, Museum of Nature and Human Activities, Hyogo. Yayoigaoka 6, Sanda, Hyogo 669-1546, Japan

**Keywords:** cpDNA, Lamiaceae, nrDNA, Phylogenetics, *Salvia akiensis*, *Salvia lutescens*

## Abstract

Both *Salvia akiensis* and *S. lutescens* (Lamiaceae) are endemic to Japan. *Salvia akiensis* was recently described in 2014 in the Chugoku (= SW Honshu) region, and each four varieties of *S. lutescens* distributed allopatrically. Among varieties in *S. lutescens*, var. intermedia show a disjunctive distribution in the Kanto (=E Honshu) and Kinki (= W Honshu) regions. Recent field studies of S. lutescens var. intermedia revealed several morphological differences between the Kanto and Kinki populations. Here, I evaluated these differences among Salvia lutescens var. intermedia and its allies with morphological analysis and molecular phylogenetic analyses of nuclear ribosomal DNA (internal and external transcribed spacer regions) and plastid DNA (*ycf1-rps15* spacer, *rbcL*, and *trnL-F*) sequences. Both morphological analysis and molecular phylogenetic analyses showed that S. lutescens var. intermedia from the Kinki region and var. lutescens were closely related to each other. However, var. intermedia from the Kanto region exhibited an association with S. lutescens var. crenata and var. stolonifera, which also grew in eastern Japan, rather than var. intermedia in the Kinki region. These results indicated that S. lutescens var. intermedia is not a taxon with a disjunctive distribution, but a combination of two or more allopatric taxa. Present study also suggested that *S. akiensis* was most closely related to *S. omerocalyx*.

## Introduction

The genus *Salvia* L. (tribe Mentheae) is the largest genus in Lamiaceae; it comprises nearly 1,000 species. *Salvia* has radiated extensively into three regions of the world: Central and South America (500 spp.), West Asia (200 spp.), and East Asia (100 spp.) (Alziar, 1988–1993). In Japan, twelve species, eight varieties, and one putative hybrid have been described since Thunberg’s (1784) first account. The genus was classified into three subgenera (subg.), including *Allagospadonopsis* Briq., *Salvia*, and *Sclarea* (Moench) Benth, (Hihara et al. 2001, Inoue 1997, Murata and Yamazaki 1993, Takano et al. 2014). Most of the taxa are endemic to Japan, with the exception of *S. japonica* Thunb., *S. nipponica* Miq. and *S. plebeia* R. Br. (Murata and Yamazaki 1993).

There are four varieties known in *S. lutescens* (Koidz.) Koidz.: var. crenata, var. intermedia, var. lutescens, and var. stolonifera (Murata 1952, Yonekura and Kajita 2003 onwards). Fukuoka and Kurosaki (1982) noticed distribution of each taxon does not overlap and clarified that the distribution of var. crenata on the Japan Sea side of Central to Northern Honshu, var. stolonifera on the Pacific side of Central Honshu, var. lutescens around the Suzuka Mountain range (Mie Pref., W Honshu), and the disjunctive distribution of var. intermedia in the Kanto (E Honshu) and Kinki regions (W Honshu) based on herbarium works.

Takano and Okada (2011) conducted molecular phylogenetic analyses of Japanese *Salvia* and found that the species were distributed among three subclades: (1) *S. plebeia* (subg. Sclarea), (2) subg. Salvia, and (3) subg. Allagospadonopsis. They also found four varieties of *S. lutescens* that did not form a monophyletic group; instead, they were dispersed among several clades in phylogenetic trees, based on both plastid DNA (cpDNA) and nuclear ribosomal DNA (nrDNA) data, and their topologies were not concordant with each other. In addition, they became paraphyletic in the phylogenetic trees based on combined cpCNA and nrDNA data (Takano and Okada 2011). Furthermore, during a recent field survey, I noticed that S. lutescens var. intermedia in the Kanto and Kinki regions had different morphological characteristics. The basal part of the anther connective was generally glabrous in the Kanto population, but it was pilose in the Kinki population. Also, in the Kanto population, the stalk of the inflorescence declinated toward the ground after flowering, and it typically became proliferous; in contrast, in the Kinki population, the inflorescence grew erect, and it was never proliferous.

Recently, a new species of Japanese *Salvia*, *S. akiensis* A.Takano, T.Sera et Kurosaki has been described from Shimane and Hiroshima Prefectures (Takano et al. 2014). At the moment, this species shows disjunctive distribution, ca. 40 km away from each, and the habitat is also very different between Hiroshima and Shimane: it grows among bamboo by roadsides and on slopes below evergreen mixed forests and plantations in Shimane (Sakoda et al. 2014), but it is found in moist, shallow soil on rock walls by streams in deciduous forests in Hiroshima (Takano et al. 2014). Therefore, it may wonder if the species be monophyletic. Takano et al. (2014) discussed relationships among *S. akiensis*, *S. isensis* Nakai ex H.Hara, *S. lutescens* and *S. omerocalyx* Hayata based on morphological characters, but molecular phylogenetic position of *S. akiensis* remains unclear.

As a step toward taxonomic revision of variety of *S. lutescens* and to confirm monophyly and phylogenetic position of *S. akiensis*, morphological and molecular phylogenetic analyses were conducted. Takano and Okada (2011) followed the Murata and Yamazaki (1993) system in which treated var. intermedia as a forma f. lobatocrenata and var. lutescens as f. lutescens, however, here I follow the Murata (1952) system (=Y-list, Yonekura and Kajita 2003 onwards), and each infraspecific taxon of *S. lutescens* is treated as a variety.

## Materials and methods

### Morphological analyses on *S. lutescens* in herbaria

Murata (1952) studied morphological variations in the plants of subgen. Allagospadonopsis in Japan and found hairiness, number or shape of leaflets, presence /absence of glandular hairs were so variable and could not be used as diagnostic characters. Diagnostic characters separated each variety of *S. lutescens* are indumentums of the basal part of the anther connective and floral color (Nakai 1950, Murata 1952). Among varieties, var. lutescens shows pale yellow flowers and pilose at the base of anther connective, var. intermedia shows deep violet corolla and pilose at the base of anther connective, var. crenata does purple corolla and glabrous base of anther connective. Floral color and indumentums of var. stolonifera is same as var. crenata, however, var. stolonifera extends its stolon after anthesis (Nakai 1950). Since it is difficult to know exact floral color by examining dry specimens, the indumentums at the base of the anther connective were observed for glabrousity in selected specimens, which bore at least several flowers. A total of 89 specimens of S. lutescens var. intermedia, including its syntypes, of the 34 specimens are from Kanto region and 55 from Kinki, were examined in the following herbaria: the Museum of Nature and Human Activities, Hyogo (Hyogo); the Kanagawa Prefectural Museum (KPM); Kyoto University (KYO); Tokyo Metropolitan University (MAK), the Osaka Museum of Natural History (OSA), and The University of Tokyo (TI) (Appendix 1). Additionally, all the specimens of S. lutescens var. lutescens including its holotype at KYO were examined on the same characters, since no information on that character is available.

### DNA extraction, PCR, and DNA sequencing

The protocols for DNA extraction, polymerase chain reaction (PCR), purification, and DNA sequencing were described previously by Takano and Okada (2011). The PCR conditions and the PCR and sequencing primers for *rbcL*, the *trnL-F* intergenic spacer region of cpDNA (*trnL-F*), and the internal transcribed spacer (ITS) region of nuclear ribosomal DNA (nrDNA) were also described previously by Takano and Okada (2011). To amplify in the *ycf1-rps15* spacer region found in cpDNA (*ycf1-rps15*), *5711f* and *rps15r* (both from Drew and Sytsma 2011) were used as PCR primers in PCR assays, and ETS-*bdf1* (Drew and Sytsma 2011) and 18S-E (Baldwin and Markos 1998) were used to amplify the external transcribed spacer (ETS) sequence from 18S-26S ribosomal DNA. The four PCR primers were also used for sequencing. The PCR conditions for amplifying the two loci were: denaturation at 95 °C for 3 min, followed by 40 cycles at 95 °C for 30 s, 54 °C for 30 s, and 72 °C for 30 s; and a final extension at 72 °C for 5 min.

### Sequence alignment and phylogenetic analysis

Raw sequence data were assembled and edited manually, with BioEdit software (ver. 7.2.5 Hall 1999)

DNA sequences were aligned with the CLUSTALW 1.83 software package, with default settings and multiple alignments (Thompson et al. 1994). Alignments of the *rbcL*, *trnL-F*, and *ycf1-rps15* sequences of cpDNA, and the ITS and ETS sequences of nrDNA were combined. Gaps were deleted.

**Figure 1.**
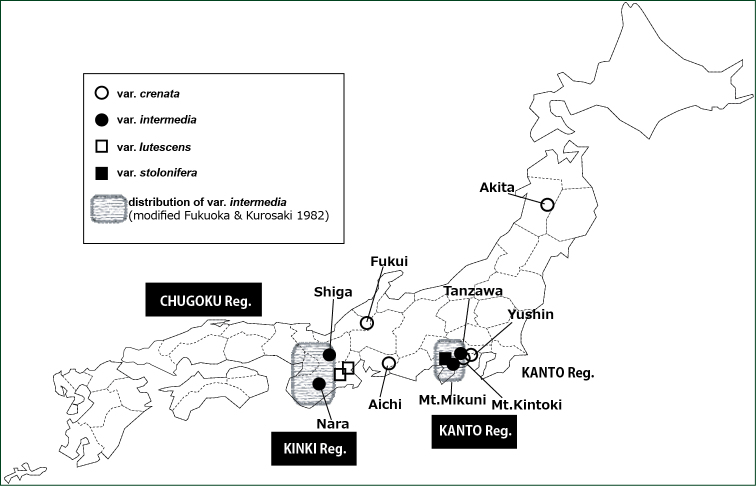
Map of Japan shows the sites where *Salvia lutescens* taxa were sampled. Open circle = var. crenata, filled circle = var. intermedia, open square = var. lutescens, filled square = var. stolonifera. The areas encircled with dotted lines show the Kinki and Kanto regions, as indicated.

Compared to Takano and Okada (2011), the ETS (Baldwin and Markos 1998) and *ycf1-rps15* of cpDNA (Dong et al. 2015) were newly sequenced for all samples. Further, two individuals of *S. akiensis* and three of S. lutescens var. intermedia, three of S. lutescens var. crenata, and one each of *S. isensis*, S. japonica var. japonica, S. lutescens var. lutescens, and *S. plebeia* were newly added for the analysis. The sampling sites of *S. lutescens* group were shown in Fig. 1. A total of 36 individuals of *Salvia* were used, including all the *Salvia* taxa from Japan and one Taiwanese *Salvia* (*S. arisanensis* Hayata). *Salvia polystachya* M. Martens et Galeotti and *S. plebeia* were selected as outgroup; the former species belonged to clade II sensu Maria and Classen-Bockhoff (2014), which became a sister to group IV and contained the East Asian *Salvia*; the latter species became a sister to a species of the subgenus Allagospadonopsis and *Salvia* (Hu 2015). Materials, accession numbers for the sequences, vouchers, and references to the literature are presented in Table 1. The sampling sites for the varieties of *S. lutescens* are shown in Fig. 1.

**Table 1.** Taxa, Genbank accession number, and voucher specimens/references used in this study. Newly sequecned data are shown bold.

Name	Pop. Code	*rbcL*	*trnL-F*	*ycf1-rps15*	ETS	ITS	Voucher / References
*S. akiensis* A.Takano, T.Sera et Kurosaki	HIR (Hirohsima Pref.)	**LC124176**	**LC124188**	**LC060529**	**LC060825**	**LC060729**	A.Takano and N.Kurosaki with T.Sera 130606-1(Hyogo)
	S1(Shimane Pref.)	**LC124177**	**LC124189**	**LC060530**	**LC060826**	**LC060728**	M.Sakoda et al. 1 (Hyogo,KYO)
*S. arisanensis* Hayata		AB295063	AB295074	LC060531	LC060827	AB295085	Sudarmono and Okada (2007)
*S. glabrescens* (Franch. et Sav.) Makino							
var. glabrescens	FS (Wakasa, Fukui)	AB541134	AB541148	**LC060532**	**LC060828**	AB541120	Takano and Okada (2011)
var. repens (Koidz.) Kurosaki	KY (Kyoto)	AB295064	AB295075	**LC060533**	**LC060829**	AB295086	Sudarmono and Okada (2007)
*S. isensis* Nakai ex Hara	MIE	AB266221	AB266231	**LC060534**	**LC060830**	AB266241	Sudarmono and Okada (2007)
	AICHI	**LC124178**	**LC124190**	**LC060535**	**LC060831**	**LC060730**	A-200933 (living material at Hiroshima Bot.Gard. Originally from Owariasahi city, Aichi Pref.)
*S. japonica* Thunb.							
f. albiflora Hiyama		AB266220	AB266230	**LC060536**	**LC060832**	AB260240	Sudarmono and Okada (2007)
f. japonica	Osaka	AB266219	AB266229	**LC060537**	**LC060833**	AB266239	Sudarmono and Okada (2007)
f. japonica	Gotenba	**LC124179**	**LC124191**	**LC060538**	**LC060834**	**LC060731**	A.Takano 140806-5 (Hyogo)
f. longipes (Nakai) Sugimoto		AB266218	AB266228	**LC060539**	**LC060835**	AB266238	Sudarmono and Okada (2007)
*S. koyamae* Makino		AB541128	AB541142	**LC060540**	**LC060836**	AB541114	Takano and Okada (2011)
*S. lutescens* Koidz.							
var. crenata (Makino) Murata	AICHI	AB266223	AB266233	**LC060541**	**LC060837**	AB266243	Sudarmono and Okada (2007)
	Yushin	AB353202	AB353198	**LC060542**	**LC060838**	AB353206	Takano and Okada (2011)
	Akita	**LC124180**	**LC124193**	**LC124205**	**LC124201**	**LC124203**	Y. Horhii, S. Nishida et al. 2015026 (Hyogo)
	Fukui	**LC124181**	**LC124194**	**LC124204**	**LC124200**	**LC124202**	A.Takano 150702-1a (Hyogo)
var. intermedia (Makino) Murata	Nara	**LC124182**	**LC124195**	**LC060544**	**LC060840**	**AB295097**	Sudarmono and Okada (2007)
	Shiga	**LC124183**	**LC124196**	**LC060546**	**LC060842**	**LC060735**	A.Takano 140821-1 (Hyogo)
	Mt.Mikuni	**LC124184**	**LC124197**	**LC060547**	**LC060843**	**LC060733**	A.Takano 140806-4 (Hyogo)
	Tanzawa	**LC124185**	**LC124198**	**LC060548**	**LC060844**	**LC060734**	A.Takano 140622-2 (Hyogo)
var. lutescens Koidz.	MIE	AB266222	AB266232	**LC060549**	**LC060845**	AB266242	Sudarmono and Okada (2007)
	Aoyama	**LC124186**	**LC128192**	**LC060550**	**LC060846**	**LC060737**	a201241 (living material at Hiroshima Bot.Gard. Originally from Aoyama Kogen, Mie Pref.)
var. stolonifera G.Nakai		AB541139	AB541153	**LC060551**	**LC060847**	AB541125	Takano and Okada (2011)
*S. nipponica* Miq.							
var. nipponica	TOKU (Tokushima)	AB541132	AB541146	**LC060552**	**LC060848**	AB541118	Takano and Okada (2011)
	KUMA (Kumamoto)	AB541127	AB541141	**LC060553**	**LC060849**	AB541113	Takano and Okada (2011)
var. kisoensis K.Imai	NAK	AB541136	AB541150	**LC060554**	**LC060850**	AB541122	Takano and Okada (2011)
*S. omerocalyx* Hayata							
var. omerocalyx	HI (Hidaka, Hyogo)	AB353204	AB353196	**LC060555**	**LC060851**	AB353200	Takano and Okada (2011)
	Hyogo (Yabu, Hyogo)	AB353205	AB353197	**LC060556**	**LC060852**	AB353201	Takano and Okada (2011)
var. prostrata Satake		AB541138	AB541152	**LC060557**	**LC060853**	AB541124	Takano and Okada (2011)
*S. pygmaea* Matsum.							
var. pygmaea		AB295072	AB295083	**LC060558**	**LC060854**	AB295094	Sudarmono and Okada (2007)
var. simplicior Hatus. ex T.Yamaz.		AB541140	AB541154	**LC060559**	**LC060855**	AB541126	Takano and Okada (2011)
*S. ranzaniana* Makino		AB287373	AB287374	**LC060560**	**LC060856**	AB287375	Sudarmono and Okada (2007)
*S. × sakuensis* Naruh. et Hihara		AB541129	AB541143	**LC060561**	**LC060857**	AB541116	Takano and Okada (2011)
Outgroup							
*S. plebeia* R.Br.	KIZU	AB295073	AB295084	**LC060563**	**LC060858**	AB295095	Sudarmono and Okada (2007)
	KAMI	**LC124187**	**LC124199**	**LC060562**	**LC060859**	**LC060738**	A.Takano and N.Kurosaki 090607-2 (Hyogo)
*S. Polystachya* M.Martens et Galeotti		AY570435	JF301399	JF289067	JF301334	JF301356	Drew and Systma (2011)

The incongruence length difference (ILD) test (Farris et al. 1994) was used to evaluate congruence between the chloroplast and the nuclear data sets. 100 replications were performed using PAUP*4.010b (Swofford 2002). As the ILD test (P < 0.01) suggested incongruence between the two datasets, and the topologies also exhibited discordance, I performed separate analyses for the cpDNA and the nrDNA data. Maximum Likelihood (ML) and Bayesian inference (BI) were used. Nucleotide substitution model parameters were determined for each partition by gene was evaluated with KAKUSAN 4.0 (Tanabe 2007), and the corrected Akaike information criterion (AICc) (Sugiura 1978) was used for model selection. For the cpDNA partitions KAKUSAN suggested the HKY85 (*rbcL*) and GTR+G (trn*L-F*, *ycf1-rps15*spacer) models, and the HKY85 model for ETS and GTR+G model for ITS for the nrDNA partitions. The ML analyses were completed using TREEFINDER version March 2011 (Jobb et al. 2004). A replicated (500 iterations) partitioned analysis was performed with bootstrap (1000 rounds) using AICc separated model for nrDNA dataset and AICc proportional model for cpDNA dataset. Bayesian evolutionary analysis using partitioned datasets were run in BEAST v.1.8.3 (Drummond et al. 2012, Heled and Drummond 2010) with 20 million Markov Chain Monte Carlo (MCMC) iterations, under an uncorrelated relaxed clock (Drummond et al. 2006), Yule process of speciation with a random starting tree for each partition. Convergence of the chains was checked using the program Tracer 1.6 (Rambaut et al. 2014). High effective sample sizes were observed for all parameters (posterior ESS values > 200 for the combined analyses). Maximum clade credibility trees with divergence times means and 95% highest probability densities (HPDs) were produced using Tree Annotator (Drummond et al.2012).

## Results

### Morphological characteristics

**Figure 2.**
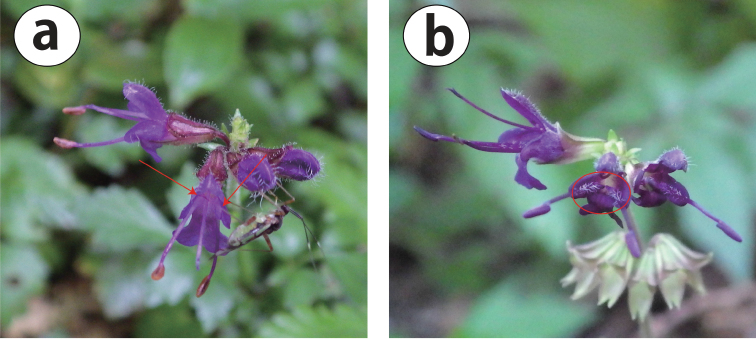
Photographs of S. lutescens var. intermedia flowers. **a** Flower of *A. Takano 140806-4-2* (Hyogo), from Mt. Mikuni, Susono-shi, Shizuoka Pref. (Kanto region). Arrows indicate the base of the anther connective. No hairs are visible **b** Flower of *A. Takano 140813-1* (Hyogo), from Mt. Yamatokatsuragi, Gose-shi, Nara Pref. (Kinki region). The red open circle indicates the base of the anther connective. White hairs are visible.

Among the 89 specimens of S. lutescens var. intermedia examined, 52 specimens from the Kinki region were pilose at the base of the anther connective (Fig. 2), and no specimens from the Kanto region shared this characteristic (Appendix 1). Ten specimens collected from the Kanto region had at least one, but less than 10 hairs. Twenty-four specimens from the Kanto region (Fig. 2) and three specimens from the Kinki region (*Y.Kato s.n.* [KYO], *T.Kobayashi 23369* [KYO], and *A.Takano 140821-1* [Hyogo]) were glabrous at the base of the anther connective. However, a duplicate of *T.Kobayashi 23369* (KYO) examined at Hyogo was pilose at the base of the anther connective (Appendix 1).

Totally, 18 specimens of S. lutescens var. lutescens were deposited at KYO and examined, 13 of these had pilose at the base of the anther connective (Appendix 1). Four of these had no flowers, and only one specimen, *M.Hara s.n.*, collected from Mt. Takami, Maze-Mura, Iinan-gun, Mie Pref. showed glabrousity.

### Phylogenetic positions of Japanese taxa in the genus *Salvia*

A likelihood analysis using the concatenate cpDNA datasets (*rbcL*+*trnL-F*+*ycf1-rps15* spacer) for 36 individuals in 23 taxa resulted in a ML tree with –lnL = 5295.264. The ML and Bayesian trees had similar topology; the Bayesian maximum clade credibility tree is shown with ML bootstrap (ML-BS) and Bayesian posterior probability (BI-PP) in Figure 4. The Japanese and Taiwanese species of subg. Allagospadonopsis formed a well supported clade (ML-BS/BI-PP, 100/0.97). Two subclades were found in the subg. Allagospadonopsis clade: (1) *S. japonica* + *S. pygmaea* + one *S. akiensis* + *S. arisanensis* + five individuals of *S. lutescens* in E Japan subclade, and (2) one *S. akiensis* (S1), two *S. isensis*, *S. lutescens* in Kinki + *S. ranzaniana* + two *S. lutescens* in the Kanto region + *S. omerocalyx*. The latter group of taxa, minus the *S. omerocalyx* (Hyogo), consisted of a strongly supported subclade, with high ML-BS/BI-PP values (98/0.99). *S. lutescens* in E Japan were scattered between both subclades, but the *S. lutescens* in the Kinki region consisted a cluster with the weak support (--/0.70).

A concatenate nrDNA datasets (ETS+ITS) yielded a ML tree with –lnL = 3789.114. The ML and Bayesian trees had similar topology; the Bayesian maximum clade credibility tree is shown with ML-BS and BI-PP in Figure 5. The Japanese and Taiwanese species of subg. Allagospadonopsis formed a well supported clade (ML-BS/BI-PP, 100/1.00). There were four subclades in the *Allagospadonopsis* clade: (1) *S. lutescens* group in E Japan + *S. isensis* (ML-BS/BI-PP, --/0.69), (2) *S. lutescens* in Kinki + *S. ranzaniana* (ML-BS/BI-PP, 61/0.57), (3) *S. arisanensis* + *S. omerocalyx* + *S. akiensis* (ML-BS/BA-PP, 76/0.99), and (4) one S. lutescens var. crenata + *S. japonica* + *S. pygmaea* (ML-BS/BA-PP, 58/0.97). Thus, *Salvia lutescens* and its allies apparently became polyphyletic. *Salvia ranzaniana* became a sister group to *S. lutescens* in the Kinki region but the ML-BS /BA-PP support was weak (61/0.57). *Salvia isensis* became a sister group to *S. lutescens* in the Kanto region with strong ML-BS/BA-PP support (86/1.00). *Salvia akiensis* formed a strongly supported subclade with *S. omerocalyx* group (89/1.00).

## Discussion

**Figure 3.**
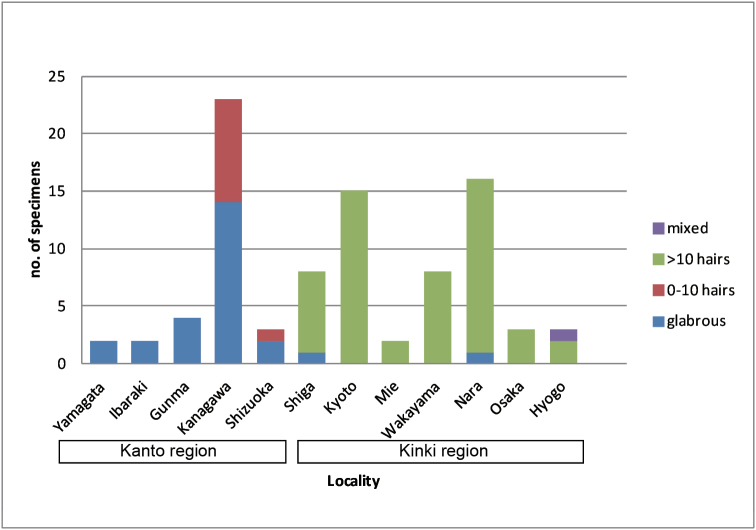
A graph shows the number of specimens examined indumentums at the base of anther connective.

**Figure 4.**
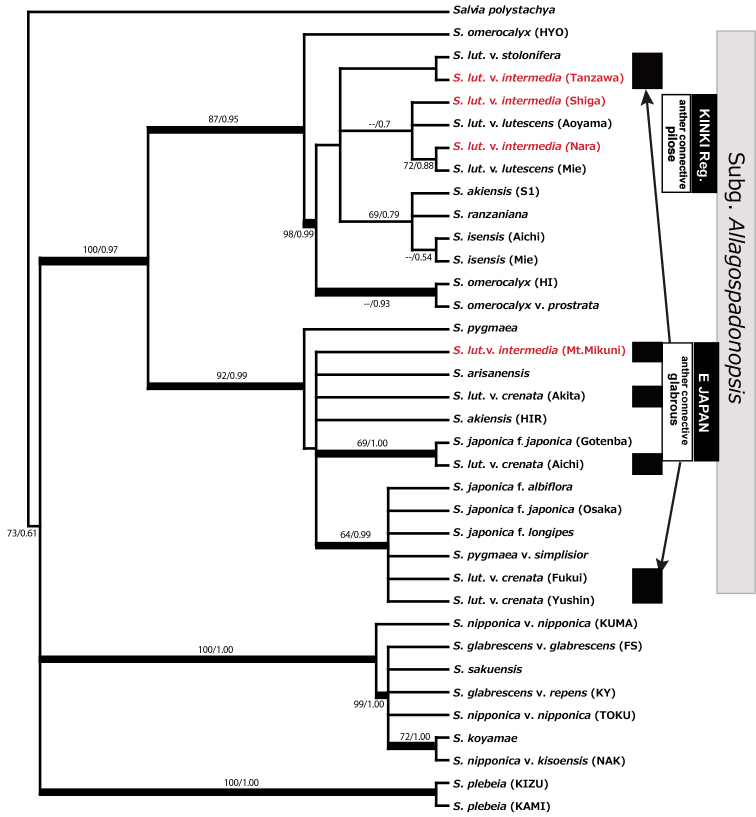
The Bayesian maximum clade credibility tree derived from plastid DNA (concatenate dataset of *rbcL*, trn*L-F*, *ycf1-rps15*). ML-bootstrap/Bayesian PP numbers are shown near the corresponding branch. Thick lines denote a clade that was strongly supported, with ML- bootstrap and/or Bayesian-PP greater than 95 %. ML: maximum likelihood; PP: posterior probability.

**Figure 5.**
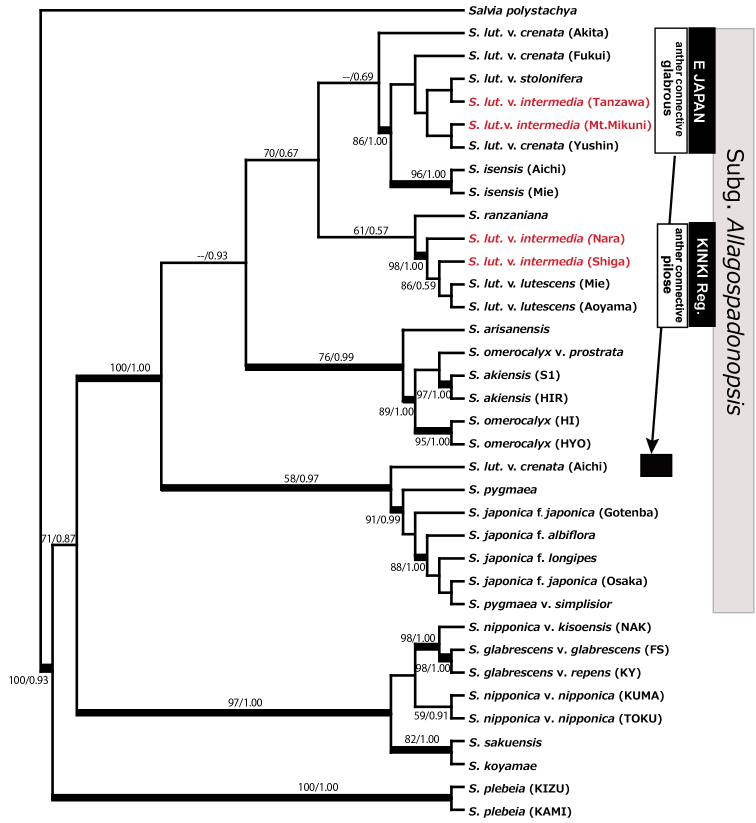
The Bayesian maximum clade credibility tree derived from nuclear ribosomal DNA (concatenate dataset of ETS and ITS). ML-bootstrap/Bayesian-PP numbers are shown above or below the corresponding branch. Thick lines denote a clade that was strongly supported with ML-bootstrap and/or Bayesian-PP values greater than 95 %. ML: maximum likelihood; PP: posterior probability.

This study suggests that S. lutescens var. intermedia is polyphyletic. Four individuals of var. intermedia, two from the Kanto and two from the Kinki region fell into different subclades in both molecular phylogenetic trees using cpDNA and nrDNA datasets, although the two from the Kinki region were always in the same subclade (Figs 4, 5). The plants of var. intermedia from the Kanto region (Tanzawa and Mt.Mikuni) fell into the same subclade in the nrDNA tree together with var. crenata, var. stolonifera, and *S. isensis* whereas they fell into different subclades in the cpDNA tree. Such a contradiction might indicate that var. intermedia from the Kanto region have multiple origins, or might have originated via hybridization or introgressive gene flow between nighbouring taxa (e.g., Sudarmono and Okada 2007). The discordance between nr DNA and cpDNA data is common in the mint family (Trusty et al. 2004, Moon et al. 2010, Drew and Sytsma 2013, Deng et al. 2015), and chloroplast-based phylogeny often does not reflect their morphological relationships, which can be explained by chloroplast capture (Rieseberg and Soltis 1991). Morphological analysis also supports the contention that var. intermedia is polyphyletic, as the specimens of var. intermedia studied showed in the indumentums at the base of the anther connective: pilose in the plants from the Kinki region, and glabrous in the plants from the Kanto region (Fig. 3).Therefore it is clear that var. intermedia from the Kinki region and the taxon from the Kanto region are different entities, suggesting that var. intermedia is not a taxon that shows disjunctive distribution, but is instead admixture of two or more biological entities. Additionally, as mentioned in introduction, after flowering the stalk of the inflorescence becomes declinate to ground and usually proliferous in case of the plants from the Kanto region, but never become declinate in the plants from the Kinki region. The indumentums at the base of anther connective is effective to select pollinators to avoid intrusion of insects which could not be effective pollinators (R.Classen-Bockhoff pers. Comm..) However, pollinators of var. intermedia in the Kinki and the Kanto region are not different (=Bombus (Diversobombus) diversus diversus, some Halictidae, and Syrphidae. Takano 2017). Habitat is also similar: half-shaded, on mesic soils along streamlet on the forest floor of deciduous forests. They might have begun to be diverged from each other after long geographical isolation.

On the contrary, present morphological and molecular phylogenetic analyses indicated that S. lutescens var. lutescens and var. intermedia from the Kinki region are closely related to each other. In molecular phylogenetic analysis, they formed a cluster in both cpDNA- and nrDNA trees, though ML-BP/BI-PP support was not strong in cpDNA tree. The morphological study revealed var. lutescens is pilose at the base of the anther connective: therefore, S. lutescens var. intermedia in the Kinki region share the same morphological status with var. lutescens. The distribution of var. lutescens is very near to that of var. intermedia in the Kinki region (Mie, Shiga, Nara Prefs.), although var. lutescens and populations of the Kinki regions of var. intermedia have never been found to grow together.

Salvia lutescens var. intermedia in the Kanto region may be more closely related to var. crenata and var. stolonifera. Murata (1952) mentioned that the base of anther connective is glabrous in var. stolonifera and var. crenata. The present study revealed that var. intermedia in the Kanto region shares this character with those two taxa. Salvia lutescens var. intermedia in the Kanto region formed a strongly supported sucblade with var. *crenata*, var. stolonifera and *S. isensis* in nrDNA phylogenetic tree. In the cpDNA phylogenetic tree, S. lutescens var. intermedia from the Kanto region (Mt.Mikuni) was included in the subclade containg *S. akiensis*, *S. japonica*, S. lutescens var. crenata, and *S. pygmaea* whereas S. lutescens var. intermedia (Tanzawa) formed a subclade with var. stolonifera and was included in the subclade containing *S. akiensis*, *S. omerocalyx*, *S. ranzaniana*, and S. lutescens var. intermedia from the Kinki + *S. isensis*. These findings suggest a close relationship among var. crenata, var. stolonifera, and var. intermedia from the Kanto region. Var. intermedia from the Kanto region may belong to var. stolonifera and var. crenata. The identity of var. intermedia and other varieties of *S. lutescens* are needed to be re-evaluated, and further study is necessary towards revision of varieties of *S. lutescens*.

The phylogenetic analyses also suggest that *S. akiensis* comprises a monophyletic group, as indicated by nrDNA tree, and that most of the species allied to *S. akiensis* was the *S. omerocalyx* group. *Salvia akiensis* and *S. omerocalyx* group comprised a subclade in nrDNA (ML-BS/BI-PP: 89/1.00). These two taxa did not form a subclade in cpDNA, but it may be of introgression/chloroplast capture /hybridization as mentioned above. In contrast, *S. akiensis* and *S. omerocalyx* share following characters: bearing the largest flowers among species in the subg. Allagospadonopsis, flower from May to June, and exhibit gynodioecy (Takano 2013; Takano et al. 2014). These characters are assumed to be symapomorph.

## Appendix 1

Specimens examined *Salvia lutescens* var. *intermedia. And* var. lutescens.

Salvia lutescens var. intermedia

**Specimens glabrous at base of anther connective** (27 sheets)

**KANTO Region. Yamagata Pref.**: Mt. Kushigata, *S.Kigawa s.n.*, July 10, 2001 (KPM *NA0151444, NA015445*). **Ibaragi Pref.**: Tsukuba, *C.Owatari s.n.*, July 8, 1895 (**syntype**, TI); Ibidem, *C.Owatari s.n.*, July 25, 1895 (**syntype**, TI). **Gunma Pref.**: Mt. Akagi, *H.Hara s.n.*, July 12, 1928 (TI); Akagi, Shikisimadori, Chubu, *H.Hara s.n.*, 11 July 1928 (TI), Kouzuke, Tone, Yunogoya-daira, *H.Hara s.n.*, 13 July, 1928(TI); Joshu, Tone-gun, Tokura, *S.Saito 145* (TI). **Kanagawa Pref.**: Hakone, *S.Ohkubo s.n.*, July 26, 1881 (**syntype** TI); Ibidem, *unknown collector*, July 26, 1881 (**syntype**, TI); ibidem, *S.Tamaki s.n.*, July 14, 1914 (TI); Minami pass, Hakone, *T.Makino 62582* (KYO); Yoduku, Yamakita-cho, *T.Katsuyama et al.*, July 23, 2005 (KPM *NA0124794*); Mikuniyama-rindo, Hakone, *Inoue et al.*, June 18, 1998 (KPM *NA0112995*); Tanzawa-Ohyama, *T.Nishio 1489* (KPM); Summit of Mt. Kintoki, Hakone, *S.Kigawa s.n.*, July 3, 1980 (KPM *NA1020531*); Ishigoya-Ochiai, Kiyokawa-mura, *H.Takahashi 20563* (KPM); Ishigoya-Ochiai, Kiyokawa-mura, *H.Takahashi 20565* (KPM); Minesaka Pass to Myojin Pass, Yamakita-cho, *S.Mori 20536* (KPM); Kaminokawa, Tsukui-machi, *S.Kigawa 20559* (KPM); Kurokura, Yamakita-machi, *M.Furuse 45371* (KPM); Ogawadani, Yamakita-cho, *A.Takano 140622-2* (Hyogo). **Shizuoka Pref.**: Mt.Mikuni, Fukayoshi, Susono-shi, *A.Takano 140806-4-1* (Hyogo); ibidem, *A.Takano 140806-4-2* (Hyogo).

**KINKI Region. Nara Pref.**; Yoshino, Yamato, *Y.Kato s.n.*, Aug. 8. 1936 (KYO). **Hyogo Pref.**:Taki-gun, Nishiki-cho, *T.Kobayashi 23369* (KYO). **Shiga Pref.**: Ikadachi-tochu, *A.Takano 140821-1* (Hyogo).

**Specimens showed one to several hairs at base of anther connective** (10 sheets)

**KANTO Region. Pref.Sizuoka**: Mt. Higane, Prov. Izu, *S. Shimazu s.n.*, July 18, 1920(KPM);

**Pref.Kanagawa**: Sirogane rindo, Yugawara-cho, *Y.Hasegawa 14263* (KPM); Ogawadani-rindo, Yamakita-cho, *T.Katsuyama s.n.*, Aug. 22, 1995 (KPM *NA0100397*); Marudake, Hakone, *M.Tashiro s.n.*,July 18, 1956 (KPM *NA0157166*); Tounomine, Hakone, *T.Deguchi 80495* (KPM); Tougadake, Yamakita-cho, *S.Kigawa s.n.*, July 3, 1980 (KPM *NA1020531*); Ishigoya-Ochiai, Kiyokawa-mura, *H.Takahashi 20564* (KPM), Mt.Ohmuro, Tsukui-machi, *S.Kigawa s.n.*, June 10, 1979 (KPM *NA1020566*); Hayatogawa Rindo, Tsukui-machi, *S.Kigawa 20541* (KPM), Youkizawa, Yamakita-machi, *S.Kigawa 20567* (KPM).

**Specimens which showed long pilose at base of anther connective** (52 sheets)

**KINKI Region. Kyoto Pref.**: Rakuhoku, Ohara, Otonashi W.F., *S.Hajacava s.n.*, Aug. 1896 (TI), Kyoto, Ohara, *T.Tsuyama s.n.*, Sep.7, 1934 (TI); Kiyotaki-Takao, Ukyo-ku, Kyoto, *S.Tsugaru & T.Takahashi 26448* (KYO); Mt.Hyotankuzure-yama, near Ohara, *G.Nakai 5401* (KYO); ibidem, *G.Nakai s.n.*, July 25, 1951 (KYO); Kadono-gun, Nakagawa-mura, *M.Tagawa 887* (KYO, two sheets); Maesaka Takanomine to Shimosugisaka, *S.Okamoto s.n.*, July 14, 1932 (KYO), Bodai W.F., Nakagawa, *G.Nakai 6305* (KYO); Mt. Kibune, *unknown collector*, June 28, 1921 (KYO); Kyoto-shi, Nakagawa to Bodai no Taki, *M.Hutoh 10515* (OSA); ibidem, *M.Hutoh 9264* (OSA); ibidem, *M.Hutoh 10528* (OSA); ibidem, *M.Hutoh 3465* (OSA); Mt.Hiei-san, *S.Tanaka s.n.*, June 30,1932 (OSA). **Hyogo Pref.**: Taki-gun, Nishiki-cho, *T.Kobayashi 23369* (Hyogo); Youtakuji, Sanda, *T.Makino 62583* (KYO); Moshi, Sanda, *A.Takano 140813-1* (Hyogo). **Nara Pref.**: Yamato, Sanjo-ga-dake to Gyojagaeri, *Y.Momiyama s.n.*, July 16, 1955(TI) (three sheets), Ibidem, *H.Hara s.n.*, July 16, 1955 (TI) ; ibidem, *T.Kobayashi 30611* (OSA); near the temple Kongo, Kashiwagi, Yamato, *K.Kondo s.n.*, June 8, 1928 (TI); Kosei River, Tenkawa Village, *K.Seto 44248* (OSA); Mt. Omine to Mt.Sanjogadake, *H.Hara 4683* (TI); en route from Wasamata hut to Mt.Nihon-dake, Kamikitayama-mura, *M.Okamoto 1966* (OSA); Mt.Daifugendake, *T.Kodama 10833* (OSA); Shonoiwaya-Mt.Wasamata Kamikitayama-mura, *K.Kodama 14356* (OSA); Irinami, Yamato, *S.Sakaguchi s.n.*, June, 1930 (KYO); Mt.Ohmine, *S.Sakaguchi s.n.*, Aug. 4, 1931 (KYO); enroute from Mt.Sanjo to Mt.Daihugen, *T.Kodama s.n.*, Aug.6,1959 (KYO); Mt.Sanjo, *G.Koizumi s.n.*, July 13, 1922 (KYO); Mt.Yamatokatsuragi, Gose, *A.Takano 140819-1* (Hyogo). **Osaka Pref.**: Mt.Izumi-katsuragi, *S.Nakanishi s.n.*, July 30, 1968 (OSA); Ibidem, *T.Nakajima s.n.*, Aug.21, 1952 (OSA); Ibidem, *C.Satonaka s.n.*, July 12, 1981 (OSA). **Wakayama Pref.**: Ryujin-Mura, Koya, *T. Nakajima s.n.*, July 31, 1931 (two sheets, TI); Doro Hacho, *G.Nakai 5213* (KYO); Ibidem, *T.Kodama s.n.*, May 30, 1951 (OSA); ibidem, *M.Hori s.n.*, May 30, 1951 (OSA); Hidaga-gun, Ooze, *S.Sakaguchi s.n.*, July 27,1932 (KYO); Mt. Sukuyama, Katsuragi-cho, Ito-gun, *K.Seto 29839* (KYO, OSA); Mt. Kurosawa, Sayiki-mura, *Y.Ogawa s.n.*, Aug.30, 1950 (KYO). **Shiga Pref.**: Otsu, *N.Takemura s.n.*, June 1901 (**Lectotype** MAK); Omi, Tochu, *M.Togashi 1205* (TI); Tochu to Ikadachi, *M.Umebayashi 737* (KYO); Mt. Hiei, *G.Murata 11415* (KYO); Ukawa, Shiga-cho, *M.Tanimoto s.n.*, June 9, 1973 (KYO); Benzaiten to Sakamoto, Mt.Hiei, S.*Tanaka s.n.*, June 30, 1932 (KYO). **Mie Pref.**: Wada, Kiwa-cho, Minami-murogun, *H.Takahashi 21040* (KYO); Taki-gun, Miyagawa-mura, Shimomate (cult.), *K.Seto 17303* (OSA).

Salvia lutescens var. lutescens

**Specimens which showed long pilose at base of anther connective** (13 sheets)

**Mie Pref.**: Itaya, Kata, Kameyama, *S.Tsugaru & T.Sawada 34155* (KYO); Notoyama, Suzuka-gun, *T.Hattori s.n.*, Aug. 5, 1928 (KYO); Kozu-mura, Naga-gun, *G.Nakai 4772* (KYO);Shinzan kokuyu-rin, Iinan-gun, *Z. Tashiro s.n.*, 4.Aug., 1934 (KYO), Mt. Gozaisyo, *G.Koizumi s.n.*, 11 Jun. 1922 (KYO); Kozu-mura, Myouga-gun, (cult. at KYO) *G.Nakai 5402* (KYO); Ibidem, *G.Nakai 4773* (KYO); Nagaishi-dani, Mt. Kamagadake, Komono-cho, Mie-gun, *N. Fukuoka 4948* (KYO); Onsen-do, Mt. Gozaisho, *G.K. & S.F. s.n.*, June 1922 (KYO, holotype).

**Shiga Pref.**: Nasugahara, Ohara-Mura, Kouga gun, *G.Koizumi s.n.*, 2 July, 1939 (KYO), Kurotaki, Tsuchiyama-cho, Koga-gun, *T. Murase 47897* (KYO); Koga-gun, Suzuka-Pass, *H. Koyama & N. Fukuoka 55* (KYO); **Nara Pref.**: Kamide, Momomata, Mitsue-mura, Uda-gun, *K.Kawabata 9994* (KYO)

## References

[B1] Alziar G (1988-1993) Catalogue synonymique des *Salvia* L. du monde (Lamiaceae). I.-VI. Biocosme Mesogéen 5(3-4): 87–136; 6(1-2, 4): 79–115, 163–204; 7(1-2): 59–109; 9(2-3): 413–497; 10(3-4): 33–117.

[B2] Baldwin B Markos S (1998) Phylogenetic utility of the external transcribed spacers (ETS) of 18S-26S rDNA: congruence of ETS and ITS trees of *Calycadenia* (Compositae). Molecular Phylogeny and Evolution 10: 449–463. https://doi.org/10.1006/mpev.1998.0545 10.1006/mpev.1998.0545 10051397

[B3] Deng T Nie Z-L Drew BT Volis S Kim C Xiang C-L Zhang J-W Wang Y-H Sun H (2015) Does the Arcto-Tertiary biogeographic hypothesis explain the disjunct distribution of northern hemisphere herbaceous plants? The case of *Meehania* (Lamiaceae). Plos One. https://doi.org/10.1371/journal.pone.0117171 10.1371/journal.pone.0117171 PMC4319762 25658699

[B4] Dong W Xu C Li C Sun J Zuo Y Shi S Cheng T Guo J Zhou S (2015) *ycf1*, the most promising plastid DNA barcode of land plants. Scientific Reports 5: 8348. https://doi.org/10.1038/srep08348 10.1038/srep08348 PMC4325322 25672218

[B5] Doyle JJ Doyle D (1987) A rapid DNA isolation procedure for small quantities of fresh leaf tissue. Phytochemical Bulletin 19: 11–15.

[B6] Drew BT Sytsma KJ (2011) Testing the monophyly and placement of *Lepechinia* in the tribe Mentheae (Lamiaceae). Systematic Botany 36: 1038–1049. https://doi.org/10.1600/036364411X605047

[B7] Drew BT Sytsma KJ (2013) The South American radiation of *Lepechinia* (Lamiaceae): phylogenetics, divergence times and evolution of dioecy. Botanical Journal of the Linnean Society 171(1): 171–190. https://doi.org/10.1111/j.1095-8339.2012.01325.x

[B8] Drummond AJ Ho SY Phillips MJ Rambaut A (2006) Relaxed phylogenetics and dating with confidence. PLoS Biology 4: e88. https://doi.org/10.1371/jounal.pbio.0040088 10.1371/journal.pbio.0040088 PMC1395354 16683862

[B9] Drummond AJ Suchard MA Xie D Rambaut A (2012) Bayesian phylogenetics with BEAUti and the BEAST 1.7 Molecular Biology and Evolution 29: 1969–1973. https://doi.org/10.1093/molbev/mss075 22367748 10.1093/molbev/mss075 PMC3408070

[B10] Farris JS Källersjö M Kluge AG Bult C (1994) Testing significance of incongruence. Cladistics 10: 315–319. https://doi.org/10.1111/j.1096-0031.1994.tb00181.x

[B11] Fukuoka N Kurosaki N (1982) Phytogeographical notes on some species of Western Honshu, Japan 3. Shoei Junior College Annual Report of Studies 14: 29–34. [In Japanese]

[B12] Hall TA (1999) BioEdit: a user-friendly biological sequence alignment editor and analysis program for Windows 95/98/NT. Nucleic Acids Symposium Series 41: 95–98. http://brownlab.mbio.ncsu.edu/JWB/papers/1999Hall1.pdf

[B13] Heled J Drummond AD (2010) Bayesian inference of species trees from multilocus data. Molecular Biology and Evolution 27: 570–580. https://doi.org/10.1093/molbev/msp274 19906793 10.1093/molbev/msp274 PMC2822290

[B14] Hihara S Iwatsubo Y Naruhashi N (2001) A new natural hybrid of *Salvia* (Lamiaceae) from Japan, Salvia × sakuensis. Journal of Phytogeography and Taxonomy 49: 163–170.

[B15] Hu G (2015) Phylogeny of *Salvia* (Lamiaceae) in East Asia. A Doctoral Dissertation, Kunming Institute of Botany, Chinese Academy of Sciences.

[B16] Inoue K (1997) *Salvia* L. In: Shimizu T(Ed.) Flora of Nagano Prefecture, 939–941. [In Japanese]

[B17] Jobb G von Haeseier A Strimmer K (2004) TREEFINDER: a powerful graphical analysis environment for molecular phylogenetics. BMC Evolutionary Biology 4: 18 https://doi.org/10.1186/1471-2148-4-18 15222900 10.1186/1471-2148-4-18 PMC459214

[B18] Moon HK Smets E Huysmans S (2010) Phylogeny of tribe Mentheae (Lamiaceae): The story of molecules and micromorphological characters. Taxon 56(1): 74–88.

[B19] Murata G (1952) Salvia subgen. Allagospadonopsis of Japan and Formosa. Acta Phytotaxonomica et Geobotanica 16: 184–190. [In Japanese]

[B20] Murata G Yamazaki T (1993) *Salvia* L. In: Iwatsuki K, Yamazaki T, Boufford DE, Ohba H (Eds) Flora of Japan IIIa, Kodansha, 302–307.

[B21] Nakai G (1950) On *Salvia japonica* and *S. lutescens*. Acta Phytotaxonomica et Geobotanica 14: 63–66. [In Japanese]

[B22] Rambaut A Suchard MA Xie D Drummond AJ (2014) Tracer v1.6, Available from http://beast.bio.ed.ac.uk/Tracer

[B23] Rieseberg LH Soltis DE (1991) Phylogenetic consequences of cytoplasmic gene flow in plants. Evolutionary Trends in Plants 5: 65–84.

[B24] Sakoda M Noriyuki M Sato K (2014) Notes on the species of subgenus Allagospadonopsis Briq. (Lamiaceae) and the habitat in Shimane Prefecture. Bunrui 14(1): 65–68. [In Japanese]

[B25] Sudarmono Okada H (2007) Speciation process of *Salvia isensis* (Lamiaceae), a species endemic to serpentine areas in the Ise-Tokai district, Japan, from the viewpoint of the contradictory phylogenetic trees generated from chloroplast and nuclear DNA. Journal of Plant Research 120: 483–490. https://doi.org/10.1007/s10265-007-0075-2 17508127 10.1007/s10265-007-0075-2

[B26] Sugiura N (1978) Further analysis of the data by Akaike’s information criterion and the finite corrections, Communications in Statistics: Theory and Methods, A7: 13–26. https://doi.org/10.1080/03610927808827599

[B27] Swofford DL (2002) PAUP*. Phylogenetic analysis using parsimony (*and other methods), version 4.0b10.Sunderland, Massachusetts: Sinauer Associates http://www.paup.sc.fsu.edu/

[B28] Takano A Okada H (2011) Phylogenetic relationships among subgenera, species, and varieties of Japanese *Salvia* L. (Lamiaceae). Journal of Plant Research 124: 245–252. https://doi.org/10.1007/s10265-010-0367-9 20628783 10.1007/s10265-010-0367-9

[B29] Takano A (2013) Gynodioecy in *Salvia omerocalyx* Hayata (Lamiaceae). Acta Phytotaxonomica et Geobotanica 63: 149–153.

[B30] Takano A Sera T Kurosaki N (2014) A new species of *Salvia* (Lamiaceae) from Chugoku district, Japan, *Salvia akiensis* sp. nov.. Acta Phytotaxonomica et Geobotanica 65: 99–104.

[B31] Takano A (2017) Flower visitors on Japanese *Salvia*. In: Proceedings of the 16^th^ annual meeting of the Japanese Society for Plant Systematics. Kyoto, Japan, 80 pp. [In Japanese]

[B32] Tanabe AS (2007) Kakusan: a computer program to automate the selection of a nucleotide substitution model and the configuration of a mixed model on multilocus data. Molecular Ecology Notes 7: 962–964. https://doi.org/10.1111/j.1471-8286.2007.01807.x

[B33] Thompson JD Higgins DG Gibson TJ (1994) CLUSTALW: improving the sensitivity of progressive multiple sequence alignment through sequence weighting, position-specific gap penalties and weight matrix choice. Nucleic Acids Research 22: 4673–4680. PMCID: PMC308517 10.1093/nar/22.22.4673 PMC308517 7984417

[B34] Thunberg C P (1784) Salvia japonica. In: Murray JA (ed) Caroli a Linné equitis Systema vegetabilium: secundum classes ordines genera species cum characteribus et differentiis. 14: 72.

[B35] Trusty JL Olmstead RG Bogler DJ Santos-Guerra A Francisco-Ortega J (2004) Using molecular data to test a biogeographic connection of the macaronesian genus *Bystropogon* (Lamiaceae) to the New World: A case of conflicting phylogenies. Systematic Botany 29: 702–715. https://doi.org/10.1600/0363644041744347

[B36] Will M Classen-Bockhoff R (2014) Why Africa matters: evolution of Old world *Salvia* (Lamiaceae) in Africa. Annals of Botany 114: 61–83. https://doi.org/10.1093/aob/mcu081 24966353 10.1093/aob/mcu081 PMC4071099

[B37] Yonekura K Kajita T (2003) BG plants Y-list. http://ylist.info

